# Biological soil crusts inhibit seed germination in a temperate pine barren ecosystem

**DOI:** 10.1371/journal.pone.0212466

**Published:** 2019-02-20

**Authors:** Jessica A. Gilbert, Jeffrey D. Corbin

**Affiliations:** Department of Biological Sciences, Union College, Schenectady, New York, United States of America; US Geological Survey, UNITED STATES

## Abstract

Biological soil crusts (BSCs) are known to affect plants’ germination and seedling establishment in arid ecosystems, but their ecological role in more mesic climates is not so well-known. We tested the effects of moss-crusted versus uncrusted soils on seed germination dynamics in a temperate pine barren ecosystem. We conducted a 35-day laboratory assay of seed germination on moss-crusted soils versus uncrusted soils from the Albany (NY) Pine Bush Preserve. We compared total seed germination and the number of days to 50% of total germination of two herbaceous perennial forb species in each soil type. Three and five times more seeds germinated on uncrusted soil than on crusted soil for bush clover (*Lespedeza capitata*) and wild lupine (*Lupinus perennis*), respectively. Seeds of both species also germinated approximately 10 days earlier on uncrusted soil than on crusted soil. This study, and others in similar habitats, show that BSCs in mesic climates can influence germination and other early life-history stages of plants. We hope that further study of the interactions between BSCs and vascular plants in mesic climates will contribute to our understanding of the ecology of BSCs outside the arid and semiarid climates where they are more extensively studied.

## Introduction

A variety of biotic and abiotic factors can strongly influence the rates of seed germination and other early plant life stages that, in turn, influence plant population structure and community composition [1–3]. Understanding such factors, and how they vary in space and time, are critical if we are to understand ecosystem processes and resilience to disturbances [4–6].

The combined effects of abiotic and biotic influences on plant establishment have been demonstrated in habitats supporting biological soil crusts (BSCs) worldwide. BSCs are aggregations of cyanobacteria, algae, lichens, and bryophytes that form thin layers on soil surfaces [7–9]. They can play an integral role in soil stability [10, 11], soil moisture retention [12, 13], and the nitrogen cycle [14, 15]. They can also influence the vascular plant community through their impacts on seed germination and seedling survival [16]. The direction and magnitude of BSCs’ impacts on plants, however, has been shown to vary widely by plant species and crust community composition. For this reason, biocrust-plant interactions should be tested empirically rather than inferred from other studies.

BSCs have been most-often studied in arid to semi-arid ecosystems, but they can also occur in more mesic climates including the temperate and subtropical US (e.g. [17, 18–21]). BSCs in these habitats can be found in open barrens, pine barrens, pavement barrens, sand plains, and dunes, and include a diverse assemblage of algae, cyanobacteria, lichens, and bryophytes (Corbin and Thiet, *unpublished data)*. Many of these habitats are recognized as systems of high conservation value for their unique ecology and the presence of rare and threatened plants and animals (e.g. [22, 23–25]).

To date, only a few studies have examined how BSCs in these more mesic climates influence plant dynamics, and, as has been observed for BSC-plant dynamics in general, the results are system- and species-specific. BSCs in New Jersey’s Pine Barrens inhibited germination of three perennial species [21], but in a Florida sand scrub, BSCs promoted germination of three short-lived perennial herbaceous species and an annual/occasional perennial [26]. Meanwhile, BSCs in a German sand ecosystem inhibited perennial species’ germination but had either a positive effect or no effect on annual species’ germination [27]. Thiet et al. [18] found that algal and lichen-moss crusts from a coastal sand dune increased seedling survivorship and growth of a perennial grass and a woody shrub, but moss-only crusts decreased seedling performance. The range of results demonstrates that a general relationship between crusts and plant demography in such habitats is not possible.

This study contributes to the growing but still small body of research investigating the role of BSCs on early-stage plant population dynamics in mesic climates. We assessed seed germination on moss-crusted versus uncrusted (sand) substrate in an inland pine barren ecosystem in New York, USA. This is the first known examination of BSCs in such a habitat, and adds needed understanding to the ecology of BSCs in temperate ecosystems.

## Materials and methods

### Study area

**Fig 1:**
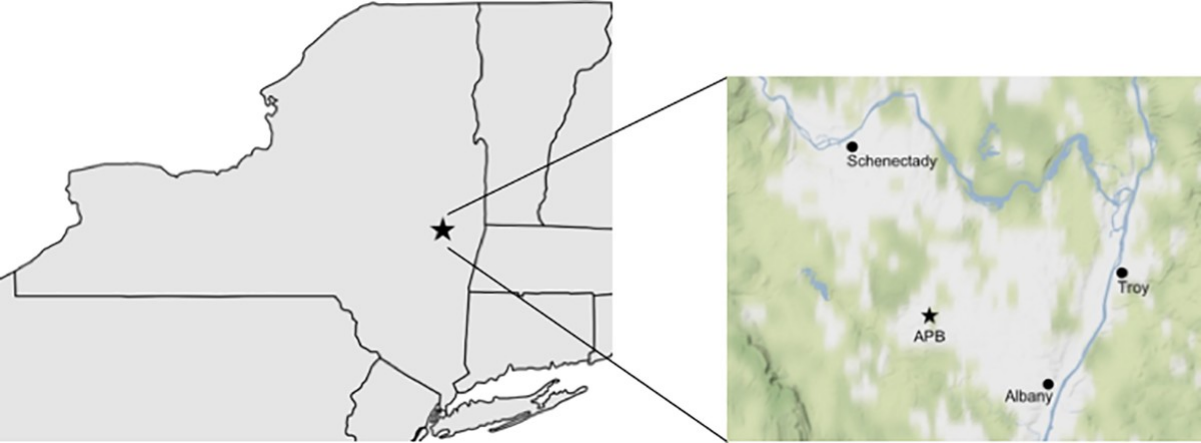
Study area at Albany Pine Bush Preserve in New York State, indicated by the stars.

Sampling permit 6-14-2017 was granted by the Albany Pine Bush Commission to Jeffrey Corbin. The Albany Pine Bush (APB) Preserve (42° 42’ N; 73° 52’ W; Fig 1) is an inland pine barren that is formed of deep, sandy, glacial outwash soils [28]. Though its cold temperate climate supports nearby eastern deciduous forest, vegetation cover at the APB is relatively sparse due to the edaphically xeric conditions and frequent fires. Native vegetation is dominated by scrub oak (*Quercus ilicifolia*), blueberry (*Vaccinium* spp.), and scattered pitch pine (*Pinus rigida)*. Areas of the APB were invaded by the non-native black locust tree (*Robinia pseudoacacia*) in the latter 20^th^ Century and subsequently restored with mechanical removal of locust stems and roots [29]. Both native pine-oak scrub and areas from which black locust trees have been removed are managed using prescribed fire [30].

Biological soil crusts are abundant in areas of the APB where fires or other recent disturbances maintain an open canopy. Vascular plants, BSCs, and uncrusted sand at the APB and other pine barren ecosystems form a spatial mosaic at the scale of 0.5–10 m^2^ per patch ([21]; personal observation). Crust composition ranges from incipient algal mats to moss-dominated crusts that also contain lichens and cyanobacteria (Corbin and Thiet, *unpublished data*). Common species include the mosses *Ceratodon purpureus* and *Polytrichum piliferum* and the lichens *Cladonia cristatella* and *C*. *gracilis* ssp. *turbinata*, and *C*. *ochrochlora*.

### Sample collection

In June 2017, we collected the substrate for our germination trials from a single site that had been cleared of the black locust tree in 2008 and subsequently replanted with a native species mix including little bluestem (*Schizachyrium scoparium* (Michx.) Nash), wild perennial lupine (*Lupinus perennis* L.) and round-headed bush clover (*Lespedeza capitata* Michx.). Soils at this site were loamy fine sands of the Colonie series. We collected mature moss-dominated BSCs by inserting 40, 3.5-cm diameter plastic Petri dishes into the soil to a depth of 2 cm. We also collected 40 dishes of bare sand, hereafter referred to as “uncrusted” samples, from the same site and within meters of our crusted samples. Crusted samples were dominated by two relatively short-statured mosses, *C*. *purpureus* and *P*. *commune*. Lichens made up a minor component, though in several dishes they increased in cover during experimental watering. Though the uncrusted samples were free of visible mosses and lichens and had a loose-sand texture, some turned green during the experiment, indicating that algae and perhaps cyanobacteria were present.

All dishes were stored in a refrigerator (4°C) for three weeks until the experiment began.

### Germination procedure

We tested germination rates of lupine and bush clover, both herbaceous perennial plants. Both species are abundant in mature pine barren habitat and also in the post-black locust removal sites such as where we collected our soil samples. Lupine and bush clover seeds were collected from wild populations at the APB in 2016. All seeds were stored in a freezer (-20°C) between cleaning and use.

Our experiment was a full factorial experiment with two soil types–moss-crusted and uncrusted–and three plant species types–lupine, bush clover, and no seeds. There were 10 replicates of each soil type x plant species treatment combination. Each dish except the no seeds treatments received 20 seeds of a single species scattered onto the surface. Seeds were not buried so as to best mimic natural wind-dispersal. During the study some seeds in the crust treatment fell into cracks in the moss canopy, and seeds in the sand treatment tended to be at least partially covered, as the experiment went on. The no seed treatments on each soil type were established as a control for seeds emerging from a potential buried seed bank; no seeds were added to this latter treatment.

Dishes were laid out randomly on a light table under 12-hour white light beginning July 2, 2017, and were rotated once a week to eliminate discrepancies in light conditions. All dishes were watered with 5 ml of water every day, which was the quantity that evenly moistened the soil or crust surface through the 2 cm crust/sand depth. Germinating seeds, judged by the emergence of the radicle, were recorded every 1–2 days for 35 days. Once a seed germinated, it was removed. We recorded the total number of seeds that germinated in each dish and the number of days it took for each dish to reach 50% of total germination (T50). T50 was not calculated for any experimental dishes in which total germination was zero.

### Data analysis

We used two-way ANOVA (R version 3.4.1) to analyze the effect of soil type (moss-crusted versus uncrusted), plant species (bush clover versus lupine), and the interaction between soil type and plant species on total germination and T50 (α = 0.05). The data were found to meet assumptions of normality and homogeneity of variance.

## Results

**Fig 2:**
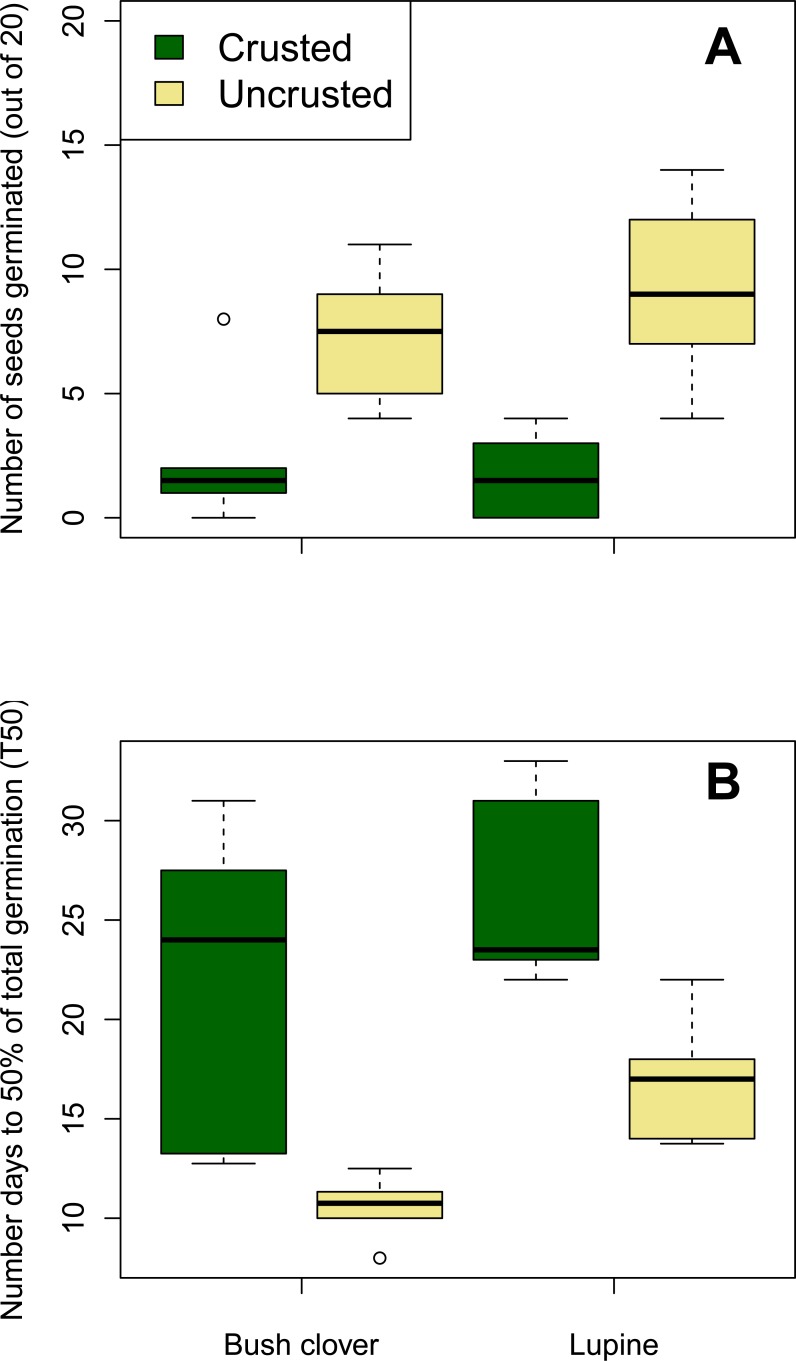
Boxplots of (A) total seed germination and (B) number of days to 50% of total germination (T50) for each soil type x plant species combination. The box in each box- and- whiskers plot represents the 25th and 75th percentiles, while the line in the middle of the box is the median (50th percentile). The top and bottom whiskers extend to the most extreme data points that are no more than 1.5 times the interquartile range from the box. The open circle is an outlier beyond the whiskers. Total seed germination was lower, and T50 was longer, on crusted soils compared to uncrusted soils. A similar number of seeds germinated for each plant species, but bush clover seeds germinated significantly faster than lupine seeds.

**Fig 3:**
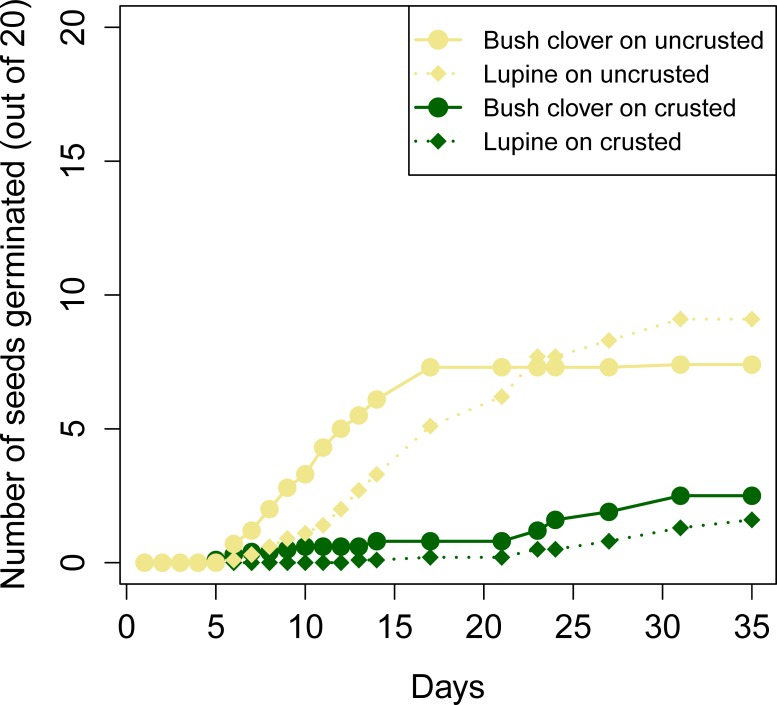
Cumulative number of germinated seeds for bush clover and lupine on each soil type during the 35-day experiment.

There was a strong effect of soil type on the number of bush clover and lupine seeds that germinated, as three and five times more bush clover and lupine, respectively, germinated on uncrusted soil than on moss-crusted soil (Table 1; Fig 2A). Seeds on uncrusted soil reached T50 approximately 10 days earlier than seeds on crusted soils. Bush clover seeds’ T50 was approximately 5 days earlier than lupine seeds’ T50 on both crusted and uncrusted soils (Table 1; Figs 2B and 3). There was no interaction between soil type and plant species on T50 (Table 1). By the end of the experiment, total germination did not differ between the two species, nor was there a significant interaction between soil type and plant species on total germination (Table 1; Figs 2 and 3).

**Table 1:** Analysis of variance comparing the effects of soil type (crusted versus uncrusted) and plant species (bush clover versus lupine), and their interaction, on total germination and time to 50% of total germination (T50).

	Total Germination			T50		
Variable	d.f.	F	p-value	d.f.	F	p-value
Soil type	**1, 36**	**54.4**	**<0.0001**	**1, 30**	**42.2**	**<0.0001**
Plant species	1, 36	0.2	0.6	**1, 30**	**13.0**	**0.002**
Soil type x Plant species	1, 36	2.4	0.13	1, 30	0.4	0.6

Significant p-values are indicated by bold text.

No seeds germinated in either our crusted or uncrusted control (“no seeds”) dishes that would indicate the presence of a stable seed bank.

## Discussion

Moss-covered BSCs at the Albany Pine Bush clearly inhibited seed germination in our experiment. Fewer seeds germinated, and they took longer to do so, on moss crusts than they did on uncrusted sand. Though some germination assays have found that BSCs can enhance rates of germination [26, 31], our findings are consistent with other studies in mesic and arid climates in which BSCs inhibited germination [18, 27, 32–35].

Though we only tested the effects of BSCs on two species, both of which were herbaceous perennial forbs, they both responded in similar ways. We saw no interaction between plant species and the type of soil for either total germination nor the time it took for germination to reach 50% of total (T50). A number of other studies have reported mixed effects of crusts on germination: crusts had negative effects on germination of some species and no effect or positive effects on others (e.g. [26, 27, 31, 34, 36]). However, until more species with a wider range of life history traits are assayed for their response to crusts in our system, we have no evidence that there are species-specific effects of BSCs on seed germination.

BSCs’ negative effects on seed germination have been explained, in part, by the physical barrier that they can create between seeds and soil [33, 35–37]. The mosses in our BSC treatments quickly absorbed the added water and thus seeds were more likely to dry out. Many of the seeds that did germinate on BSC treatments did so in cracks or gaps in the moss canopy, as has been observed in other studies (e.g. [27]). By contrast, bare soil in the uncrusted treatments provided seeds with a larger area of soil contact and potentially more moisture as described by Song et al. [37].

Our study did not consider the impact of BSCs on subsequent plant life stages, yet a variety of other studies have shown distinct effects on seedling growth and survival as well. Mosses and other crust components can limit root penetration and therefore plants’ access to moisture and nutrients [32, 33]. This can be a major source of mortality for seedlings that germinate on crust surfaces [35]. However, if seedling roots can reach through the crust to the soil, they may find enhanced growing conditions. BSCs have been shown to positively affect seedling performance, perhaps through the higher nutrient and organic content of the soil under BSCs [18, 27, 31].

BSCs’ influence on the distribution and abundance of plant species may also have broader effects on the APB ecosystem. For example, lupines host the larvae of the federally endangered Karner blue butterfly (*Lycaeides melissa samuelis*) [28]. Furthermore, both lupines and bush clover form symbioses with nitrogen-fixing bacteria, and N inputs from such plants are known to influence the nitrogen status of other components of the ecosystem [38]. Finally, BSCs in temperate [21, 39] and dryland [14] systems are known to, themselves, affect soil C, N, micronutrients, and moisture. BSCs’ role in northeastern barren and dune ecosystems is worthy of further study.

Our study is evidence that intact BSC communities can influence the plant community of pine barren ecosystems by affecting the fate of seeds. This mechanism likely contributes to the mosaic of BSC patches interwoven with vegetated patches and could be a significant source of habitat heterogeneity. Sedia and Ehrenfeld [21] hypothesized that BSCs and vascular plants form alternative stable states in pine barren ecosystems, potentially mediated by seasonal weather conditions. Until recently, there were relatively few efforts to further explore the interactions between BSCs and vascular plants in mesic climates, and how those interactions vary over space in time (but see [18, 27, 40]). We hope that further awareness of crusts outside of the arid and semiarid climates where they are more extensively studied will encourage more exploration.

## Supporting information

S1 File Seed germination data file. (CSV)

S2 File Metadata for S1 File. Description of S1 File data organization.
(TXT)
